# The Dissatisfied Dental Colleges

**Published:** 1904-08

**Authors:** 


### Editorial.

### THE DISSATISFIED DENTAL COLLEGES.

When the Association of Dental Faculties met at Washington, D. C., June 9, as narrated in our last number, the vote to a return to a three years' course stood twenty-one in favor and twenty-four against any change. This was supposed to be final, and yet there was a feeling that possibly certain colleges, in the minority, might hold that their financial interests were so deeply involved that some effort might be made to weaken this decision.

Those who thus felt had not long to wait to see a realization of their prophetic feelings. We now hear of several resignations from that body, and rumors of more to come. One school, that failed to vote either way, is so much disturbed by the outlook that the Dean feels compelled to seek advice as to the propriety of calling a special meeting of the "Faculties" late in August.

The dissatisfaction expressed at Washington, and since, is the natural outcome of the financial stress the four years' course brought to many of the dental colleges, and it was generally predicted that if the four years was insisted upon many of the colleges would be forced to close. If this should be the result, it may be a question whether this would, or would not, be detrimental to dental education.

It is not the purpose at present to discuss this phase of the subject, but rather to call attention to the fact that, if the entire number of twenty-one should resign, it would not affect the integrity of the Association of Faculties. Its work is not dependent on numbers. It has had a distinct mission to perform ever since Dr. Winder and his colleagues called the educational bodies together, in 1884, to legislate, that eventually a higher standard of dental education might be established in all the dental schools of the country.

A dean of one of the dental colleges recently resigned had the honesty to assign as a reason for its act that the very life of the school depended upon its freedom from control and ability to be its own judge of whom to admit and whom to reject, and, further, that financial interests governed its action. Whether this is the motive of all is not at present known, but behind other ostensible reasons will be found the necessity of the self-sustaining treasury.

That resignation is the only dignified course for those schools entertaining this view is admitted, but in taking it they write down each school as an enemy to advanced dental education and must bear whatever odium falls to the lot of the school that serves mammon rather than the higher ideals.

The mistake these schools are making, in the opinion of the writer, is to suppose that this course will be to their financial interests. This portion of the subject is not pleasant to dwell upon, for nothing can be more distasteful to the true lover of a high educational standard, than to weigh it in the balance with money. This, however, is their standard of valuation, not the writer's. Viewing it, then, from their side it seems certain that, in the end, they will find they have simply dug the pit for their own destruction, and from this there can be no resurrection. The world, at no period in its history, has been kind to those who have attempted to turn the wheels of civilization backward. If these schools were a law unto themselves, there might be a period of limited prosperity coming to them, but they are not now, or ever can be, in the position occupied by the dental schools of this country in 1870. The system of control has altogether changed, and the tendency is to bind the professional schools more and more to law, and from this there is no escape.

Aside from this, there is a sentiment among the people that demands the best in education, and will not be satisfied to patronize those schools that play fast and loose with the higher standards established for the best in collegiate and professional training.

The schools that have resigned and those that propose to follow in this direction will, probably, painfully recall the work, anxiety, and expense entailed in securing membership in the Association of Faculties. The door out is always wide open for those who seek greater freedom, but it will be securely bolted and barred to prevent their return to the fold. Higher professional education has no use for prodigal sons.

The National Association of Dental Examiners will now have a duty to perform. It is not a duty devolving upon this journal to point out the way. It recognizes that this Association possesses only a moral power, but if that be used properly it can meet this crisis in dental education effectively. It will accord with the wishes of those who have stood firmly for the highest available standard, if the Association of Examiners demands all that the Association of Faculties has stood for, and also make it impossible for graduates from the schools outside of the Association to be examined by the State boards. While this may or may not have legal strength, it will, at least, place the bodies that control dental education in the best light possible before a critical world.

Dental education in America is in no danger of a collapse. In the view of the writer, at no period in its history has the view been more encouraging. Some light may become dim, some will expire, but the effulgence will be continually greater with those who maintain that dentistry needs and must have a standard of training that looks to the future and not that which revolves round the crudities of a century, good in itself, but wholly unfitted for the necessities of that future that lies before the new men and the new time,—the eternal progressive march of ideas.

